# Lewis Basicity of Nitrogen-Doped Graphite Observed by CO_2_ Chemisorption

**DOI:** 10.1186/s11671-016-1344-6

**Published:** 2016-03-08

**Authors:** Hisao Kiuchi, Riku Shibuya, Takahiro Kondo, Junji Nakamura, Hideharu Niwa, Jun Miyawaki, Maki Kawai, Masaharu Oshima, Yoshihisa Harada

**Affiliations:** Department of Applied Chemistry, the University of Tokyo, 7-3-1 Hongo, Bunkyo-ku, Tokyo 113-8656 Japan; Faculty of Pure and Applied Sciences, University of Tsukuba, Ibaraki, Japan; The Institute for Solid State Physics (ISSP), the University of Tokyo, Kashiwa, Chiba Japan; Synchrotron Radiation Research Organization, the University of Tokyo, Kashiwa, Chiba Japan; Present address: Graduate School of Pure and Applied Sciences, University of Tsukuba, Ibaraki, Japan

**Keywords:** CO_2_ adsorption, Nitrogen-doped carbon, Lewis basicity, X-ray photoelectron spectroscopy, X-ray absorption spectroscopy, Infrared reflection absorption spectroscopy

## Abstract

The characteristics of CO_2_ adsorption sites on a nitrogen-doped graphite model system (N-HOPG) were investigated by X-ray photoelectron and absorption spectroscopy and infrared reflection absorption spectroscopy. Adsorbed CO_2_ was observed lying flat on N-HOPG, stabilized by a charge transfer from the substrate. This demonstrated that Lewis base sites were formed by the incorporation of nitrogen via low-energy nitrogen-ion sputtering. The possible roles of twofold coordinated pyridinic N and threefold coordinated valley N (graphitic N) sites in Lewis base site formation on N-HOPG are discussed. The presence of these nitrogen species focused on the appropriate interaction strength of CO_2_ indicates the potential to fine-tune the Lewis basicity of carbon-based catalysts.

## Background

The problem of capturing and storing of CO_2_ as a primary greenhouse gas must be solved to alleviate global warming. Captured CO_2_ is typically utilized as a simple carbon-containing feedstock (C1) for generating industrially relevant organic molecules [1]. Numerous porous solid adsorbates such as zeolites [2], metal-organic frameworks [3], and mesoporous carbons [4, 5] have been proposed for the effective capture and processing of CO_2_. Some research groups have reported that nitrogen-doped carbon materials interact more effectively with CO_2_ than inactive carbon materials do [4–8]. Furthermore, they are also expected as promising electrocatalysts for CO_2_ and oxygen reduction reaction [8, 9]. Accordingly, the nitrogen doping of carbon materials is expected to be an effective processes to enhance CO_2_ storage capacity and CO_2_ reactivity. CO_2_ is a weak Lewis acid with an electropositive carbon atom that can detect nitrogen-doping-induced basic sites on carbon materials [10, 11]; this detection is classified as a Lewis acid/base reaction.

For practical uses of CO_2_ adsorbents or catalysts, it is important to balance effective capturing and reduction reaction. A theoretical CO_2_ adsorption mechanism has been proposed for the catalysts and capturing materials currently in use. For example, hydrogen bonds may form between the surface functional groups and the oxygen atoms of a CO_2_ molecule [6]; pyridonic nitrogen species may act as anchors for CO_2_ capture [7]; lone-pair electrons of pyridinic nitrogen may be active for CO_2_ reduction [8]. However, detailed experimental information regarding the chemical state and geometry of adsorbed CO_2_ on nitrogen-doped carbon materials is scarce, which hinders the design of effective, highly selective CO_2_ capturing materials and catalysts for CO_2_ reduction.

In this study, we investigated the adsorption properties of CO_2_ on nitrogen-doped graphite (N-HOPG), synthesized using low-energy nitrogen-ion sputtering, using X-ray photoelectron spectroscopy (XPS), angle-resolved X-ray absorption spectroscopy (XAS), and infrared reflection-absorption spectroscopy (IRAS). Using the highly graphitized model N-HOPG with selective nitrogen doping, we discuss the possible contribution of nitrogen components to the formation of Lewis base sites on N-HOPG.

## Methods

A cleaved highly oriented pyrolytic graphite (HOPG, PGCSTM, Panasonic Inc.) plate was annealed at 1000 K for 30 min in an ultra-high vacuum (UHV) (<5 × 10^−7^ Pa), sputtered with 8 × 10^13^ nitrogen ions cm^−2^ at 200 eV (equivalent to 2 % nitrogen relative to the surface carbon atoms) at 300 K using an ion gun (OMI-0730, Omegatron Inc.), and annealed again at 1000 K for 1 h for surface cleaning [12].

XPS measurements were performed at BL27SU in a third-generation synchrotron radiation facility SPring-8 using a photoelectron analyzer (PHOIBOS 150, SPECS). The typical base pressures for the measurement and preparation chambers were 4 × 10^−8^ and 3 × 10^−7^ Pa, respectively. The incident photon energy and the photoemission angle were 850 eV and 45°, respectively. The total energy resolution of the XPS was 230 meV. Samples were scanned with the incident photon beam at a rate of 0.4 μm　s^-1^ to reduce the radiation damage to the adsorbed molecules. The binding energies were calibrated using the Au 4*f*_7/2_ (binding energy = 84.0 eV) peak of an evaporated gold as a reference. The N 1*s* spectra were fitted with the Voigt function (1.2 and 0.25 eV Gaussian and Lorentzian widths, respectively) as well as background subtraction using the Shirley method. The normalized intensity of each peak was calculated by multiplying the integrated intensity and the cross section of each element. N/C and O/C ratios were calculated by dividing the normalized intensities of the N 1*s* and O 1*s* spectra by that of C 1*s* spectrum, respectively. The surface nitrogen density was estimated by an assumption that nitrogen atoms are located only at the first graphite layer of HOPG. When we take into account the attenuation of photoelectrons in the bulk region, the contribution to the photoelectron from the first layer is calculated by 1−exp(−*d*/*λ*cos*θ*) where *d* is the interlayer distance, *λ* is the inelastic mean free path, and *θ* is the photoemission angle. In the present case for the C 1*s* XPS spectrum of graphite, this value is estimated to be 0.33 where *d* is 3.354 Ǻ, *λ* is 11.77 Ǻ [13] at 561 eV of kinetic energy which is obtained by subtracting the core level of C 1*s* (~284 eV) and work function of graphite (4.6 eV) [14] from the incident photon energy *hv* = 850 eV, and *θ* is 45°. Therefore, the surface nitrogen and the adsorbed CO_2_ densities (ML) are estimated by dividing the normalized intensities of the N 1*s* and O 1*s* XPS spectra by one third of that of the C 1*s* spectrum, respectively. Here, the number of the 1 ML nitrogen atoms is defined as that of the carbon atoms at the first graphite layer (3.82 × 10^15^ atoms cm^−2^). The number of the adsorbed 1 ML CO_2_ molecules is also defined as that of the carbon atoms at the first graphite layer and to get the number of CO_2_ molecules, the estimated value from O 1*s* XPS should be divided by 2 since CO_2_ is composed of two oxygen atoms and one carbon atom.

N 1*s* and O 1*s* XAS spectra were measured by the partial electron yield (PEY) mode at BL27SU. The XAS spectra were collected by setting the angle *θ* between the incident X-ray beam axis and the surface normal to 0°, 45°, and 70°. The energy resolution of XAS had a lower limit below 100 meV.

The N-HOPG was annealed at 1000 K for 30 min in the preparation chamber to remove the initially adsorbed gas before the XPS and XAS measurements were performed. The sample was exposed to CO_2_ at 300 K for 2500 s at 5.3 × 10^−4^ Pa, corresponding to 10,000 L in volume.

IRAS measurements were performed in grazing-angle reflection geometry both before and after CO_2_ adsorption at 300 K to investigate the configuration of the adsorbed CO_2_. In order to estimate the amount of the adsorbed CO_2_, temperature-programmed desorption (TPD) was performed between 300 and 700 K, with CO_2_ adsorption at 300 K. Both the IRAS and TPD experiments were performed in the same UHV (3 × 10^−8^ Pa) chamber.

## Results and Discussion

**Fig. 1:**
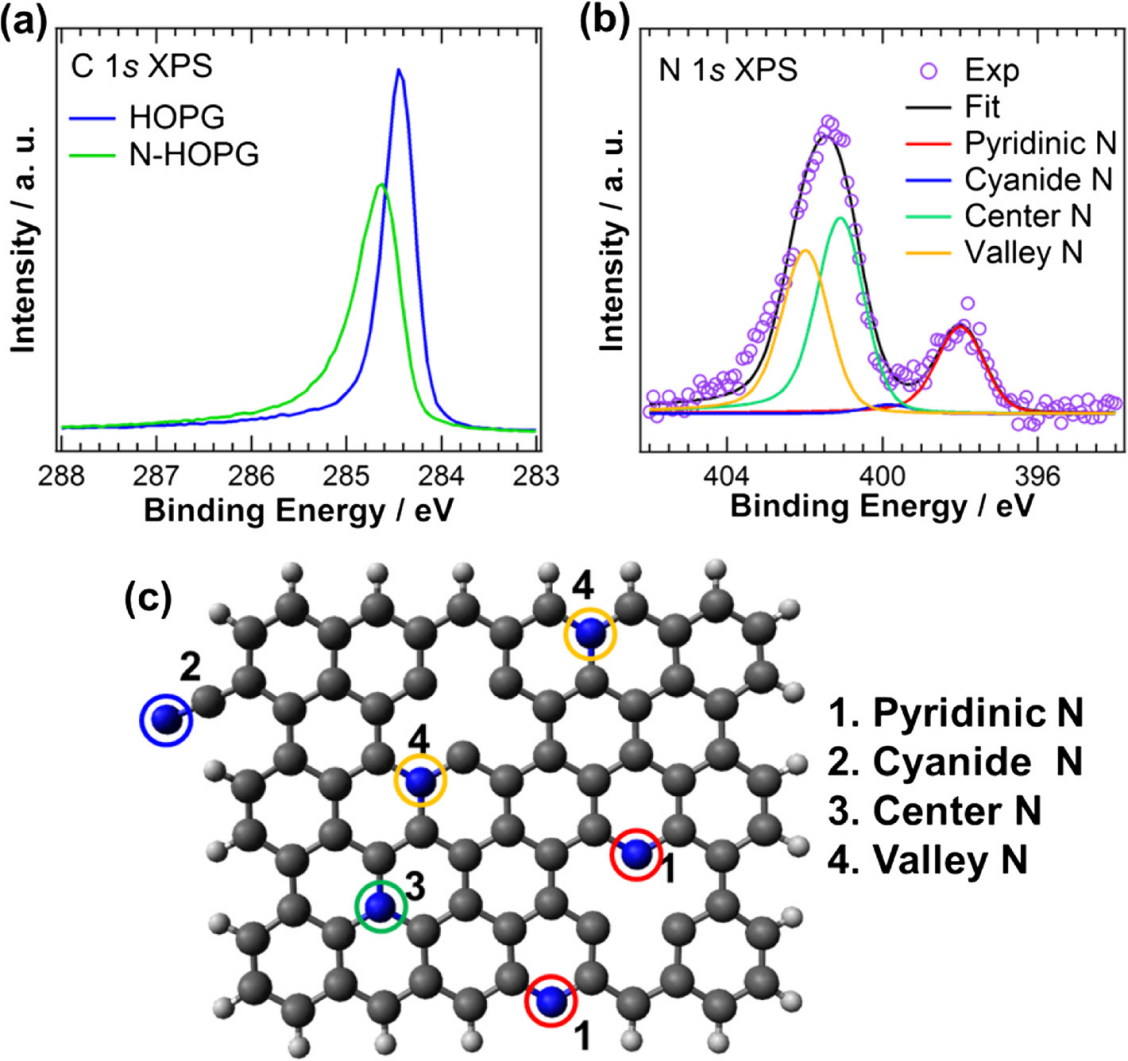
XPS spectra of HOPG and N-HOPG before CO_2_ adsorption. **a** C 1*s* XPS. **b** N 1*s* XPS. **c** A schematic of each nitrogen configuration in N-HOPG. Hydrogen, carbon, and nitrogen atoms are represented by *white*, *black*, and *blue balls*, respectively

Figure 1a shows the C 1*s* XPS spectra of HOPG and N-HOPG. The spectral intensities are normalized to the peak areas. The nitrogen ion sputtering causes the C 1*s* peak to broaden and introduces the C=N bond peak at 285.6 eV [15]. Figure 1b shows the N 1*s* XPS spectrum of N-HOPG. The N/C ratio and surface nitrogen density are calculated to be 0.0042 and 0.013 ML, respectively. The spectrum is fitted with four Voigt functions corresponding to each nitrogen component, denoted as pyridinic N (398.0 eV), cyanide N (399.9 eV), center N (401.0 eV), and valley N (401.9 eV). The terminology of the nitrogen components used hereafter is represented in Fig. 1c. Pyridinic N is connected to two carbon atoms and exists either at graphite edges or in the basal plane of graphite with a monovacancy [16]. Cyanide N has triple bonds between the nitrogen and carbon atoms [17]. Both center N and valley N are graphitic N connected to three carbon atoms, existing in the graphite basal plane, and at graphite zigzag edges or vacancy sites, respectively [18, 19]. The detailed peak assignment of each component was discussed in our previous report [12]. The estimated amount of each nitrogen component from the fitting results is summarized in Table 1; center N is the most prevalent, followed by valley N and pyridinic N.

**Table 1:** Surface nitrogen density and relative ratio of each nitrogen component

Type of nitrogen	Pyridinic N	Cyanide N	Center N	Valley N
Nitrogen density/ML	0.0024	0.0002	0.0054	0.0045
Relative ratio/%	19	2	43	36

**Fig. 2:**
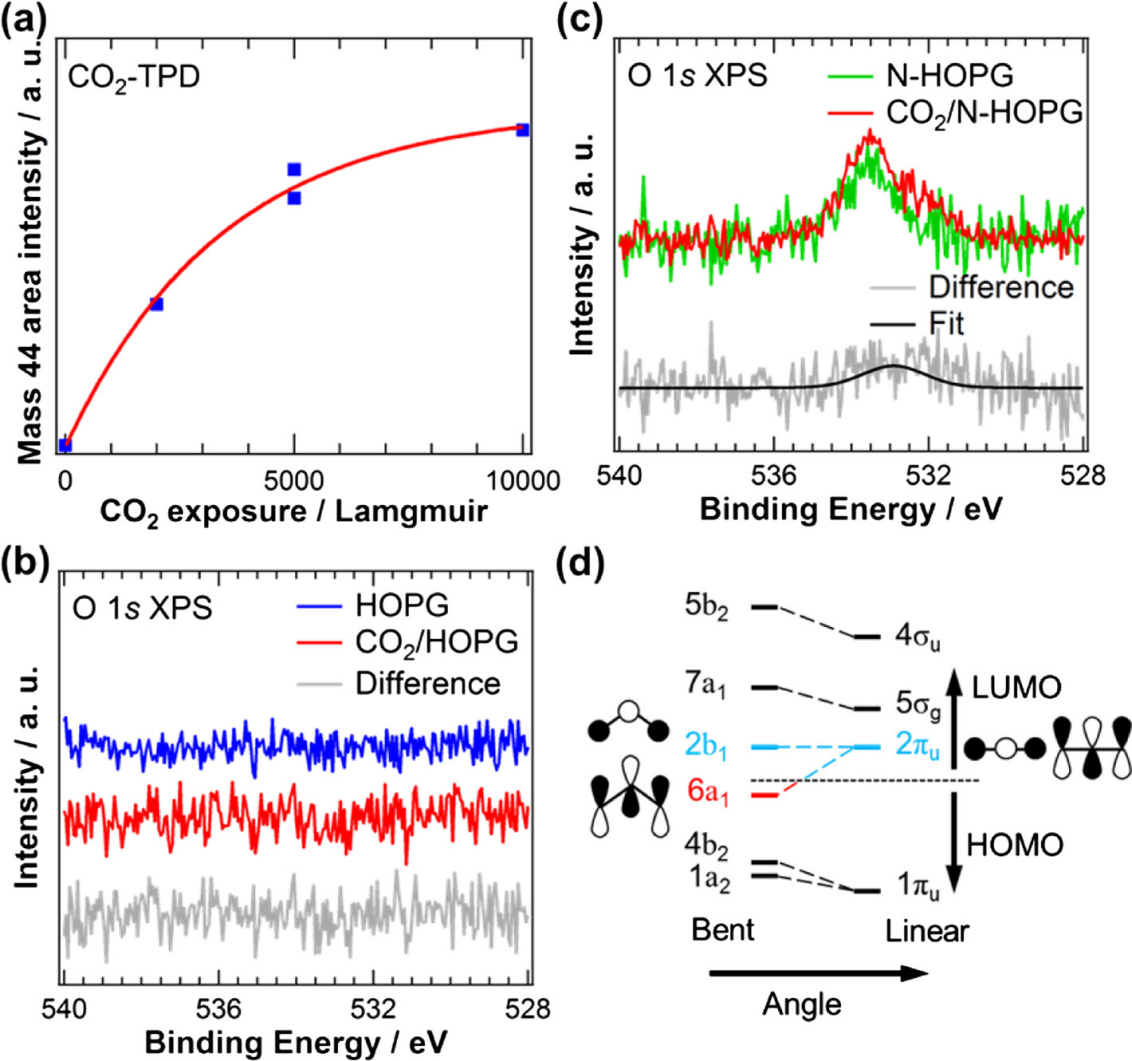
**a** The area intensity of the mass 44 (CO_2_) peak as a function of the CO_2_ exposure. O 1*s* XPS spectra of **b** HOPG and **c** N-HOPG before and after CO_2_ adsorption. **d** A schematic of the Walsh diagram of CO_2_ orbital energies in linear and bent geometries

Figure 2a shows the estimated area intensity of the mass 44 (CO_2_) peak as a function of CO_2_ exposure obtained by the TPD measurements. The saturation amount of adsorbed CO_2_ on N-HOPG is near 10,000 L; this amount was used for the XPS and XAS measurements. A clear CO_2_ desorption peak is observed between 320 and 400 K, which approaches the temperature of CO_2_ desorption for nitrogen-doped graphite model catalysts [20]. In addition, the amount of the adsorbed CO_2_ on N-HOPG is almost constant when 5000 L CO_2_ exposure was performed twice as shown in Fig. 2a, which indicates CO_2_ adsorption is reversible without poisoning. In contrast, only physisorbed CO_2_ was reported on HOPG, which desorbed at 83 K with the desorption energy of 20 kJ mol^−1^ [21]. Therefore, nitrogen doping forms CO_2_ adsorption sites at 300 K on the HOPG surface. Figure 2b, c shows O 1*s* XPS spectra of HOPG and N-HOPG before and after CO_2_ adsorption, respectively. A very small amount of oxygen species (O/C = 0.04 at.%) exists in N-HOPG before CO_2_ adsorption, as shown in Fig. 2c, which are not fully removed by annealing at 1000 K for 30 min. The difference spectrum in Fig. 2c shows an O 1*s* peak centered at approximately 533 eV caused by CO_2_ adsorption on N-HOPG. The extracted O/C ratio and the adsorbed CO_2_ density are calculated to be 0.00023 and 0.00035 ML, respectively, based on the procedure described in the “Methods” section. However, the difference spectrum in Fig. 2b of HOPG shows no peak related to adsorbed CO_2_. Table 2 summarizes the O 1*s* binding energies for physisorbed and chemisorbed CO_2_ on various substrates [22–27]; for physisorbed CO_2_, the binding energy is distributed from 534.0 to 535.8 eV, while for chemisorbed CO_2_, it ranges from 530.6 to 533 eV. The observed CO_2_ binding energy at 533 eV on N-HOPG is below that of physisorbed CO_2_ but within the range of chemisorption. The interaction strength of CO_2_ directly estimates the degree of Lewis basicity of the N-HOPG substrate. To determine the origin of this binding energy, we discuss the electronic structure of CO_2_ as presented by a Walsh diagram in Fig. 2d [28]. The lowest unoccupied molecular orbital (LUMO) of 2π_u_ is degenerated in the linear configuration, while it splits into 2b_1_ (perpendicular to the CO_2_ plane) and 6a_1_ (parallel to the CO_2_ plane) orbitals in the bent configuration. In particular, the energy position of 6a_1_ orbital sharply decreases upon bending. In the bent configuration, this low-energy 6a_1_ orbital is occupied by electrons charge-transferred from the substrate and stabilizes the adsorbed CO_2_ molecules on N-HOPG. Thus, the charge transfer from the substrate to the 6a_1_ orbital in the bent configuration weakens the strength of CO bonds, causing the O 1*s* core level shift of CO_2_ to the lower binding energy at approximately 533 eV. This result also provides evidence for the presence of Lewis base sites in the graphite system caused by the doped nitrogen.

**Table 2:** O 1*s* binding energies for physisorbed and chemisorbed CO_2_ on various substrates

Substrate	O 1*s* binding energy/eV		Ref.
	Physisorption (CO_2_)	Chemisorption (CO_2_^δ−^)	
Ni(110)	534.7	531.1	[22]
Ni(110)	534	530.6	[23]
Fe(poly)	535	531	[22]
Cr_2_O_3_(0001)	–	532.5	[24, 25]
K doped Rh(111)	534.7	532.8	[26]
K doped Mo_2_C	535.8	533	[27]
N doped HOPG	–	533	This work

**Fig. 3:**
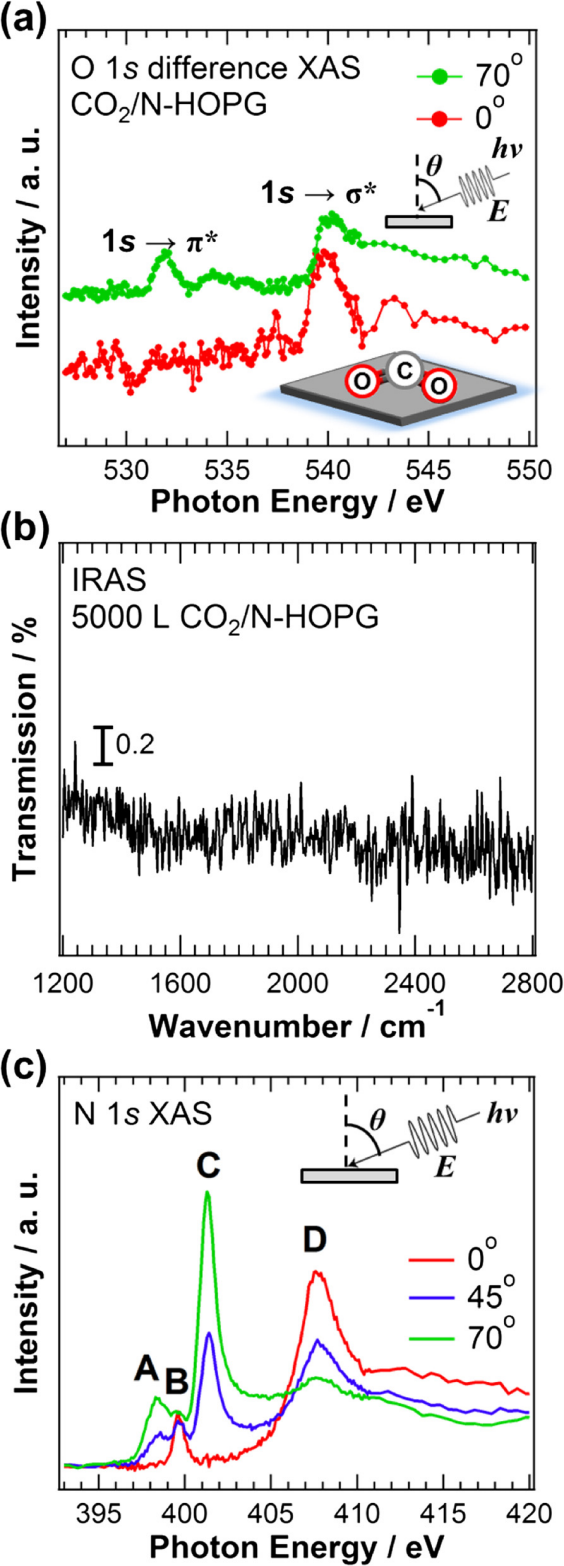
**a** O 1*s* XAS spectra of N-HOPG after CO_2_ adsorption. **b** IRAS spectrum of N-HOPG with 5000 L CO_2_ adsorption. **c** N 1*s* XAS spectra of N-HOPG before CO_2_ adsorption

In order to reveal the orientation of the adsorbed CO_2_ on N-HOPG, angle-resolved XAS was performed. Figure 3a shows the O 1*s* XAS spectra of N-HOPG after CO_2_ adsorption for different incident angles *θ*, where the background XAS profile obtained before CO_2_ adsorption is subtracted. The σ* (540 eV) and π* (532 eV) peaks are enhanced at surface normal (0°) and grazing (70°) incidence, respectively. The observed π* orbital would be derived from the 2b_1_ orbital (perpendicular to the CO_2_ plane) caused by the splitting of CO_2_ LUMO orbitals. Therefore, this polarization dependence of O 1*s* XAS indicates that the CO_2_ molecules are lying flat on the N-HOPG surface. Although the energy position of the 6a_1_ orbital sharply decreases upon bending, the energy position of the 2b_1_ orbital remains nearly constant relative to that of the 2π_u_ orbital, as shown in Fig. 2d. The observed π* peak is ~2.8 eV lower than the π* peak (2π_u_ orbital) for the gas-phase linear CO_2_ at 534.8 eV [29], mainly because of the observed chemical shift of the O 1*s* core level in XPS, as discussed above.

The IRAS spectrum in Fig. 3b also supports the above CO_2_ configuration; considering the selection rule of IRAS, the absence of peaks at approximately 1200–1300 cm^−1^ and 1600 cm^−1^, expected for the symmetric and asymmetric OCO-stretching modes of the bent CO_2_^δ−^ carboxylate with its molecular plane perpendicular to the surface [25], suggests the CO_2_ to be lying flat on the surface. Both the O 1*s* XAS spectra and the IRAS spectrum of the N-HOPG support this configuration of the adsorbed CO_2_.

Figure 3c shows the N 1*s* XAS spectra of the N-HOPG before CO_2_ adsorption for different incident angles of 0°, 45°, and 70°. The spectra are normalized according to the intensity at 430 eV. Three sharp peaks in the π* region can be assigned to pyridinic N (A), cyanide N (B), and graphitic N (center N and valley N) (C) [12, 30]. A peak at 407.5 eV corresponds to the σ* state of the nitrogen components. The intensities of the pyridinic and graphitic N change with the X-ray incident angles, indicating that these components are incorporated into the planar graphite lattice via substitution [31]. The cyanide N, however, does not show any polarization dependence, suggesting multiple directions of the C≡N configuration. Since the adsorbed CO_2_ also shows a highly oriented (lying flat) configuration, CO_2_ adsorption sites should be introduced in the vicinity of the highly oriented pyridinic and graphitic N (center N and valley N).

Based on the Lewis acid/base-interaction picture, several research groups have reported possible mechanisms of CO_2_ adsorption and reduction on nitrogen-doped carbon materials as follows: (1) hydrogen bonds forming between the carbon surface and oxygen atoms in a CO_2_ molecule [6], (2) pyridonic N species as anchors for CO_2_ capture [7], (3) a lone-pair electron located at a nitrogen atom [8], and (4) a localized electronic state of the graphite surface modified by doped nitrogen [20, 32–34]. The possibility of mechanism (1) can be excluded, because hydrogen atoms are much less prevalent in the large platelet graphite used in this study than they can be in graphene flakes. Mechanism (2) can also be excluded because pyridonic N species, located at approximately 400 eV [7], are not observed in the N 1*s* XPS spectrum. For the presence of a lone-pair electron that interacts with CO_2_, the most probable amine functional groups, located at 399.4 eV [16], are not observed because of the pyrolysis at ~1000 K in this work, but pyridinic N, which is present in this work, provides a possibility for mechanism (3) [8]. We consider that mechanism (4) is the most probable for creating the active sites for CO_2_ adsorption and reduction. The pyridinic N detected in our results may work as a Lewis base and interact with CO_2_. Indeed, recent scanning tunneling spectroscopy measurements have revealed the presence of an electronic structure just 370 meV below the Fermi level of several localized carbon atoms surrounding pyridinic N at monovacancy sites [33]. The observed valley N site is also expected to modify the electronic structure of the surrounding carbon atoms and induce a local density of states of ~200−400 meV below the Fermi level [34], which may also provide CO_2_ adsorption and reaction sites. Therefore, the interaction sites with CO_2_ in N-HOPG would be provided by the lone-pair electrons of pyridinic N or the localized π states just below the Fermi level induced by the pyridinic N and/or valley N. According to the recent report with HOPG model catalysts, pyridinic N can create Lewis base sites for CO_2_ adsorption on carbon materials [20], which is consistent with our present discussion. The clear evidence of the charge transfer from the substrate and the new information on the molecular orientations of both CO_2_ and nitrogen moieties on the graphite substrate provide great insight into the CO_2_ adsorption mechanism, and the Lewis basicity of nitrogen-doped graphite can be directly estimated by element-specific synchrotron analyses.

## Conclusions

We have investigated the adsorbed CO_2_ at 300 K on N-HOPG by XPS, angle-resolved XAS, and IRAS using a model N-HOPG synthesized by a protocol developed in a previous study. The O 1*s* binding energy of the adsorbed CO_2_ at 300 K, located at 533 eV, showed clear evidence of the Lewis basicity of N-HOPG caused by the nitrogen doped in the graphite system. The polarization dependence of the σ* and π* peaks in the O 1*s* XAS spectra, as well as the absence of expected IRAS signals, clearly indicates that the adsorbed CO_2_ lies flat on the graphite surface and that Lewis base sites are induced by the incorporation of nitrogen in HOPG. We discussed possible CO_2_ adsorption sites induced by pyridinic N and valley N species. The activity of these species as adsorption sites indicates the potential to fine-tune the Lewis basicity of a graphite substrate by controlling the prevalence of nitrogen species focused on the appropriate interaction strength of CO_2_, which can be directly estimated by the O 1*s* binding energy of the adsorbed CO_2_. The properties of fine-tuned Lewis basicity in carbon-based materials can be applied to the field of catalysis, including the use of the materials as oxygen reduction reaction or hydrogen evolution reaction sites.

## Abbreviations

CO_2_: carbon dioxide
HOPG: highly oriented pyrolytic graphite
IRAS: infrared reflection-absorption spectroscopy
LUMO: lowest unoccupied molecular orbital
N-HOPG: nitrogen doped highly oriented pyrolytic graphite
PEY: partial electron yield
TPD: temperature-programmed desorption
UHV: ultra-high vacuum
XAS: X-ray absorption spectroscopy
XPS: X-ray photoelectron spectroscopy
